# *Populus euphratica GLABRA3* Binds *PLDδ* Promoters to Enhance Salt Tolerance

**DOI:** 10.3390/ijms24098208

**Published:** 2023-05-03

**Authors:** Ying Zhang, Kexin Yin, Jun Yao, Ziyan Zhao, Zhe Liu, Caixia Yan, Yanli Zhang, Jian Liu, Jing Li, Nan Zhao, Rui Zhao, Xiaoyang Zhou, Shaoliang Chen

**Affiliations:** 1 Key Laboratory of Forest and Flower Genetics and Breeding of Ministry of Education, College of Biological Science and Technology, Beijing Forestry University, Beijing 100083, China; zying@bjfu.edu.cn (Y.Z.); ykx0303@126.com (K.Y.); zzyan913@163.com (Z.Z.); liuz6415@163.com (Z.L.); caixiayan2019@163.com (C.Y.); zhangyl@bjfu.edu.cn (Y.Z.); liujian20170703@163.com (J.L.); lijing70747@163.com (J.L.); zhaonan19880921@126.com (N.Z.); ruizhao926@126.com (R.Z.); zhouxiaoyang@bjfu.edu.cn (X.Z.); 2 Guangdong Provincial Key Laboratory of Silviculture, Protection and Utilization, Guangdong Academy of Forestry, Guangzhou 510520, China; yaojun990@126.com

**Keywords:** *Populus euphratica*, *PeGLABRA3*, phospholipase D, phosphatidic acid, ROS, Na^+^/H^+^ anti-transporter, DNA pull-down, luciferase reporter assay, virus-induced gene silencing, electrophoretic mobility shift assay, noninvasive micro-test technique, NaCl

## Abstract

High NaCl (200 mM) increases the transcription of *phospholipase Dδ (PLDδ)* in roots and leaves of the salt-resistant woody species *Populus euphratica*. We isolated a 1138 bp promoter fragment upstream of the translation initiation codon of *PePLDδ*. A promoter–reporter construct, *PePLDδ-pro*::GUS, was introduced into Arabidopsis plants (*Arabidopsis thaliana*) to demonstrate the NaCl-induced *PePLDδ* promoter activity in root and leaf tissues. Mass spectrometry analysis of DNA pull-down-enriched proteins in *P. euphratica* revealed that PeGLABRA3, a basic helix–loop–helix transcription factor, was the target transcription factor for binding the promoter region of *PePLDδ*. The PeGLABRA3 binding to *PePLDδ*-pro was further verified by virus-induced gene silencing, luciferase reporter assay (LRA), yeast one-hybrid assay, and electrophoretic mobility shift assay (EMSA). In addition, the *PeGLABRA3* gene was cloned and overexpressed in Arabidopsis to determine the function of PeGLABRA3 in salt tolerance. *PeGLABRA3*-overexpressed Arabidopsis lines (OE1 and OE2) had a greater capacity to scavenge reactive oxygen species (ROS) and to extrude Na^+^ under salinity stress. Furthermore, the EMSA and LRA results confirmed that PeGLABRA3 interacted with the promoter of *AtPLDδ* in transgenic plants. The upregulated *AtPLDδ* in *PeGLABRA3*-transgenic lines resulted in an increase in phosphatidic acid species under no-salt and saline conditions. We conclude that PeGLABRA3 activated *AtPLDδ* transcription under salt stress by binding to the *AtPLDδ* promoter region, conferring Na^+^ and ROS homeostasis control via signaling pathways mediated by PLDδ and phosphatidic acid.

## 1. Introduction

Phospholipase D (PLD) hydrolyzes membrane phospholipids to produce a secondary signaling molecule, phosphatidic acid (PA), to mediate salt stress signaling in plants [1,2,3,4,5,6,7,8,9,10,11,12,13]. Various types of PLD, particularly PLDα, PLDδ, and PLDζ, have been shown to mediate ionic and reactive oxygen species (ROS) homeostasis under salt stress. Cucumber (*Cucumis sativus*) CsPLDα-produced PA alleviates the salt damage in tobacco plants by accumulating osmoprotective compounds, maintaining Na^+^/K^+^ homeostasis and scavenging ROS [14,15]. *AtPLDα*-overexpressed poplars increase the activity of superoxide dismutase (SOD), catalase (CAT), and peroxidase (POD) under NaCl treatment [16]. *PLDa1* interacts with the downstream Cys-rich receptor-like kinase 2 (CRK2) to promote Arabidopsis (*Arabidopsis thaliana*) callose deposition and regulate plasmodesmal permeability during salt stress [17]. Phopholipase Dζ1 is considered necessary for regulating ion transport and antioxidant response in terms of the *pldζ1* mutant response to salt stress [18]. Cotton (*Gossypium hirsutum*) GhPLDδ and PA are involved in regulating plant defense against salt stress [19]. We found that *PePLDδ*, isolated from the salt-resistant woody species *Populus euphratica*, was able to increase PA content and improve salt tolerance by regulating K^+^/Na^+^ and ROS homeostasis in Arabidopsis [20]. *PLDδ* genes are induced by NaCl in rice (*Oryza sativa*) [5] and soybean (*Glycine max*) [6]; however, the NaCl-induced transcriptional regulation of *PLDδ* in poplar trees and its relevance to salinity tolerance have been less investigated.

The basic helix–loop–helix (bHLH) transcription factors have various functions in regulating plant growth, development, and stress response [21,22,23,24,25,26,27,28,29,30]. The expression of members of the bHLH gene family in response to salt stress has been characterized in *Solanum lycopersicum* [31], common bean (*Phaseolus vulgaris*) [32], and finger millet (*Eleusine coracana L.*) [33]. Moreover, NaCl was found to upregulate a number of *bHLH* genes in Arabidopsis [34], rice [35], and sugar beet (*Beta vulgaris*) [36]. The overexpression of *AtbHLH92* increases tolerance to salt stress in Arabidopsis [37]. *AtbHLH112* confers salinity tolerance through an increased ability for proline accumulation and ROS scavenging [34]. Ectopic expression of sugar beet *BvBHLH93* [36], grape (*Vitis vinifera*) *VvbHLH1* [38], and apple (*Malus xiaojinensis*) *MxbHLH18* [39] in Arabidopsis enhanced the activities of antioxidant enzymes and salt-responsive genes. Furthermore, tobacco (*Nicotiana tabacum*) transcription factor *bHLH123* was found to mediate the rapid accumulation of ROS and the response to salinity during the early stages of stress [40]. bHLH transcription factors may also improve ionic homeostasis in salinized plants. For example, *BvBHLH93* ectopic expression lines accumulated less Na^+^ but more K^+^ than the wild type (WT) [36]. bHLH122 regulates *AtKUP2* expression to confer K^+^/Na^+^ homeostasis in Arabidopsis plants [41]. The bHLH transcription factors AtMYC2 and AtbHLH122 regulate *AtNHX1* and *AtNHX6* to mediate the salinity response in Arabidopsis plants overexpressing mangrove (*Avicennia officinalis*) *AoNHX1* [42]. In rice seedlings, *OsbHLH035* confers recovery from salt stress through the ABA-independent activation of *OsHKT1s*, thus preventing Na^+^ over-accumulation and toxicity in aerial tissues [35]. bHLH transcription factors regulate the expression of genes involved in salt stress tolerance by binding to their E-box and GCG-box motifs [34]. *bHLH106* locates a branching point in the abiotic stress response network by interacting directly with the G-box in genes conferring salt tolerance on plants [43]. We also found that the *P. euphratica PePLDδ* promoter regions harbored a cis-acting G-box; however, it is unknown whether the bHLH transcription factor can mediate *PePLDδ* expression under salt stress.

Here, we report that PeGLABRA3, a bHLH transcription factor, regulates *PePLDδ* expression and plays an important role in salt stress tolerance. The binding of PeGLABRA3 to the *PePLDδ* promoter (*PePLDδ*-pro) was identified by DNA pull-down and mass spectrometry assay, virus-induced gene silencing (VIGS), luciferase reporter assay (LRA), yeast one-hybrid assay (Y1H), and electrophoretic mobility shift assay (EMSA). We investigated the salt-induced expression of *PePLDδ*-pro in root and leaf tissues in transgenic *A. thaliana*. *PeGLABRA3* was isolated from *P. euphratica* and transferred into *A. thaliana* under the control of the *CaMV35S* promoter. We examined the *AtPLDδ* expression and major PA species in WT and *PeGLABRA3*-transgenic Arabidopsis. Our data showed that the *PeGLABRA3* domain can bind to the G-box in the *AtPLDδ* promoter regions, thus exerting regulation of the gene transcription. AtPLDδ produced a higher abundance of specific PA species, such as 34:2 (16:0–18:2), 34:3 (16:0–18:3), 36:4 (18:2–18:2), and 36:5 (18:2–18:3), contributing to the maintenance of ionic and ROS homeostasis in *PeGLABRA3*-transgenic plants under NaCl stress.

## 2. Results

### 2.1. NaCl-Induced Transcription of PePLDδ in P. euphratica

**Figure 1:**
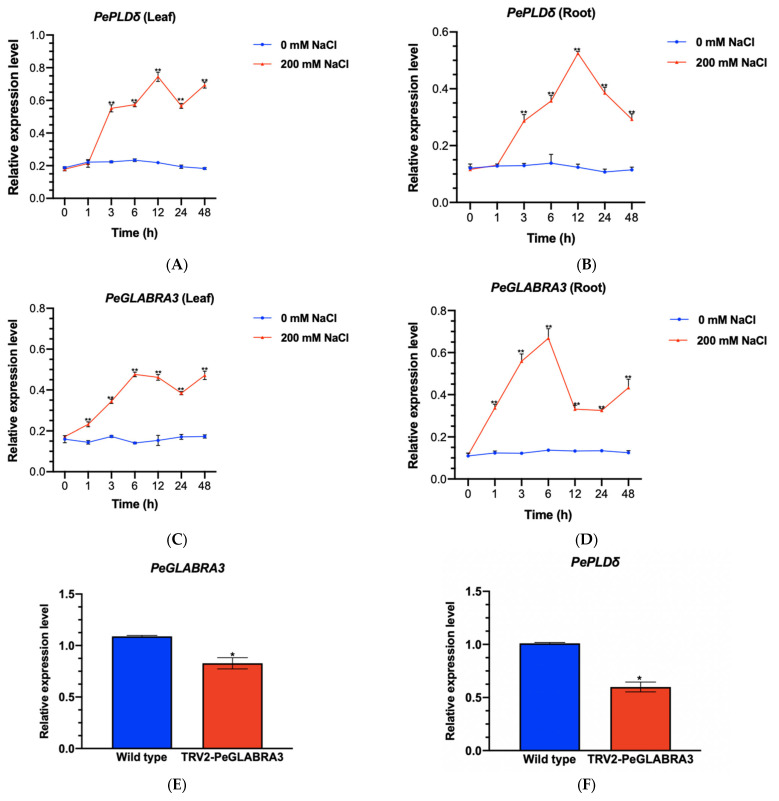
Transcription of *PeGLABRA3* and *PePLDδ* in salt-stressed *Populus euphratica*. (**A**–**D**) *PePLDδ* and *PeGLABRA3* transcription in NaCl-stressed *P. euphratica*. *Populus euphratica* seedlings were exposed to 0 or 200 mM of NaCl for 48 h. Leaves and roots were sampled at 0, 1, 3, 6, 12, 24, and 48 h for RT-qPCR analysis. (**E**,**F**) *PeGLABRA3* and *PePLDδ* transcription in *PeGLABRA3*-silenced *P. euphratica*. *Agrobacterium* carrying the TRV1 empty vector and TRV2-*PeGLABRA3* vector were injected into *P. euphratica* leaves. After 45 days of virus-induced gene silencing (VIGS), the *P. euphratica* seedlings were subjected to 200 mM of NaCl for 24 h. Leaves were sampled from TRV-infected *P. euphratica* for RT-qPCR analysis. *Populus euphratica PeACT7* was used as an internal reference. Primers for *PeGLABRA3*, *PePLDδ*, and *PeACT7* are shown in Supplementary Table S1. Each point (**A**–**D**) or column (**E**,**F**) represents the average of three independent replicates, and the error bar represents the standard error of the mean. * *p* < 0.05, ** *p* < 0.01.

*Populus euphratica PLDδ* has previously been shown to increase salt tolerance in Arabidopsis plants [20]. In this study, quantitative real-time PCR (RT-qPCR) was used to determine the salt-induced transcription of the *PePLDδ* gene in the roots and leaves of *P. euphratica* seedlings. The expression of *PePLDδ* significantly increased after 3 h of NaCl treatment (200 mM), reaching peak levels at 12 h (Figure 1A,B). Then, *PePLDδ* remained at relatively high levels in the hours following salt treatment (24–48 h), although a decline was observed in the roots (Figure 1A,B).

### 2.2. PePLDδ Promoter Cloning and Analysis

**Figure 2:**
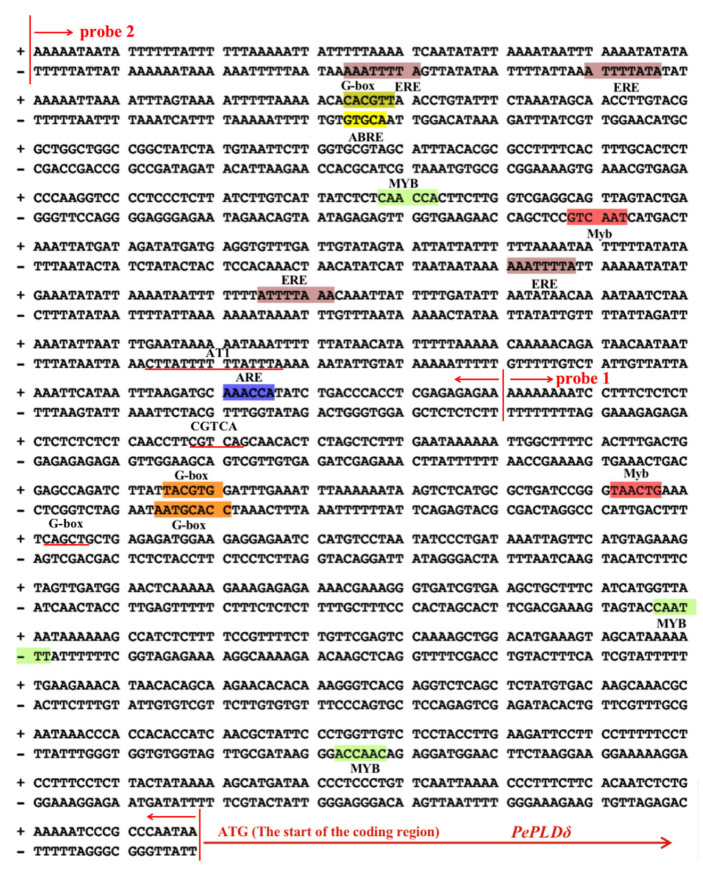
Promoter sequence analysis of *Populus euphratica PePLDδ*. The *P. euphratica PePLDδ* promoter was isolated via PCR amplifications, and the primer sequences used for *PePLDδ* promoter isolation are shown in Supplementary Table S1. The sequences of the two probes (probe 1 and probe 2) that were amplified and subsequently used for *PePLDδ* promoter isolation are shown. Different colors represent the predicted cis-acting elements in the promoter region.

The upstream regulatory region was characterized to determine its importance for the salt-induced expression of *PePLDδ*. The 1138 bp promoter fragment of *PePLDδ* was sequenced and analyzed using the PLACE and PlantCARE databases (Figure 2). Its promoter sequence was found to contain multiple cis-acting elements, including an ethylene-responsive element, a cis-acting element involved in the abscisic acid responsiveness (ABRE motif), a cis-acting regulatory element essential for the anaerobic induction (ARE), a cis-acting regulatory element involved in MeJA responsiveness (CGTCA motif), a gene element involved in drought and ABA signaling response (MYB), AT1 motif (part of a light-responsive module), and a G-box (a cis-regulatory element involved in light response) (Figure 2).

### 2.3. NaCl Activates the PePLDδ Promoter in Root and Leaf Tissues

**Figure 3:**
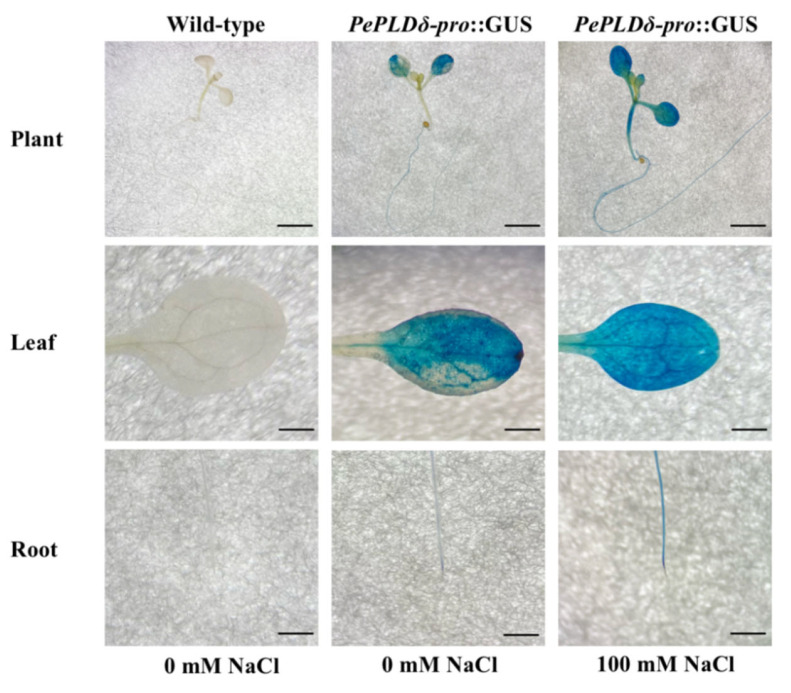
GUS histochemical assays. The promoter of *PePLDδ* was combined with the *β-glucuronidase* gene (GUS) and transformed into Arabidopsis. Ten-day-old wild type seedlings and *PePLDδ*-pro::GUS transgenic plants (T3 generation) were treated with 0 or 100 mM of NaCl for 24 h. Roots and leaves were sampled for GUS histochemical analysis. Scale bar = 500 μm.

The *PePLDδ*-pro::GUS fusion was constructed and transferred to *A. thaliana*. GUS activity was observed in the root tip, mesophyll, and vein tissues (Figure 3). Furthermore, an increase in GUS activity in root and leaf tissues was observed following NaCl treatment (100 mM, 24 h). Therefore, the salt-enhanced expression of *PePLDδ* in *P. euphratica* was due to the promoter activity.

### 2.4. Transcription Factor Identification by DNA Pull-Down

**Figure 4:**
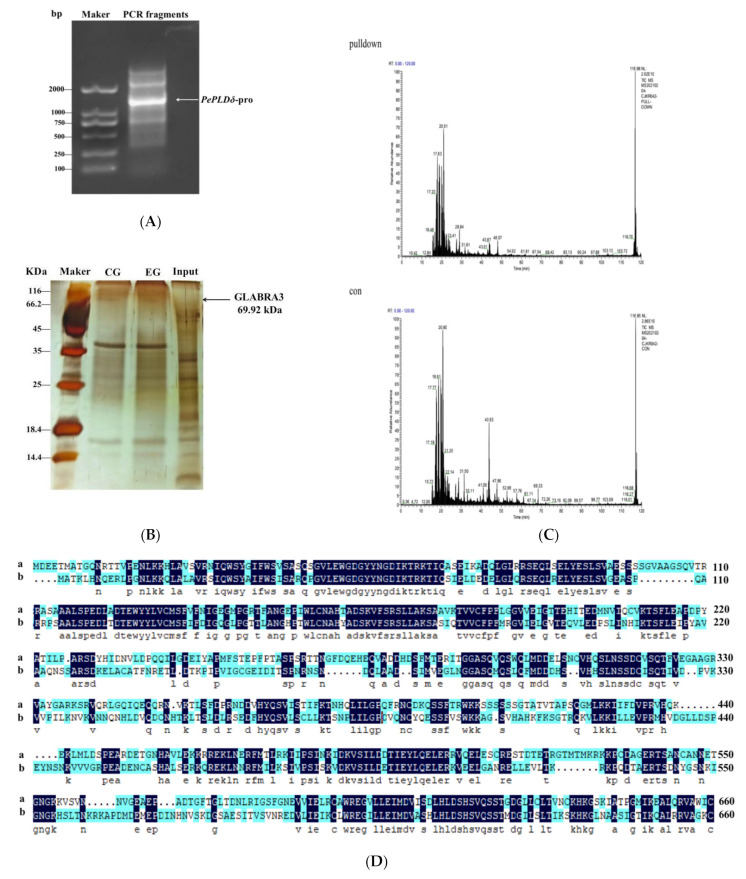
DNA pull-down and mass spectrometry analysis. (**A**) PCR amplification of the *PePLDδ* probe. The *PePLDδ* promoter fragment was amplified by PCR, cloned, and sequenced. The probes were biotin-labeled and purified with a gel extraction kit and used for DNA pull-down. (**B**) DNA pull-down-enriched proteins. The biotin-labeled DNA and nucleic acid were incubated with beaver magnetic beads to form a DNA magnetic bead complex. Then, the DNA magnetic bead complex was incubated with protein extracts to form the protein-DNA-magnetic bead complex. The DNA pull-down-enriched proteins were separated using SDS-PAGE denaturing polyacrylamide gel electrophoresis and stained with a silver staining kit (Sigma-Aldrich). CG: control group; EG: experimental group. (**C**) Mass spectrometry analysis of DNA pull-down-enriched proteins. The green marks represent the matching isotope peaks. (**D**) The amino acid sequence identified by DNA pull-down covered and matched with that of PeGLABRA3; a, the protein sequence identified by DNA pull-down; b, the PeGLABRA3 protein sequence. Black shading indicates identical amino acid residues and blue shadings indicate conserved amino acids, respectively. The obtained protein sample was analyzed with mass spectrometry (Ultraflex III mass spectrometer, Bruker, Germany). The database used for protein determination was Uniprot_*Arabidopsis thaliana* (https://www.uniprot.org/uniprotkb?query=Arabidopsis%20thaliana, accessed on 13 August 2020), Uniprot_*Populus euphratica* (https://www.uniprot.org/uniprotkb?query=Populus%20euphratica, accessed on 16 December 2020), and Uniprot_*Populus trichocarpa* (https://www.uniprot.org/uniprotkb?query=Populus%20trichocarpa, accessed on 16 December 2020). By comparison of protein type and amino acid sequence available in the NCBI database, Arabidopsis GLABRA3 was most similar to the obtained protein, and the basic helix–loop–helix (bHLH) transcription factor GLABRA3 (GL3) was identified as a target transcription factor for binding the promoter region of *PePLDδ*. Con: control group.

The identification of regulatory transcription factors for salt-induced *PePLDδ* was performed by DNA pull-down and mass spectrometry. Specific DNA probes were designed based on the sequence of the *PePLDδ* promoter region and labeled with desulfurizing biotin, which can bind to streptavidin coupled to magnetic beads (Figure 4A). Then, the protein extract was incubated with the magnetic bead DNA probe to obtain the *PePLDδ*-interacted transcription factor (Figure 4B). The target DNA probe–protein complex was obtained and identified by mass spectrometry (Figure 4C). Based on a comparison of protein type and amino acid sequence available in the NCBI database, the amino acid sequence identified by DNA pull-down covered and matched with that of PeGLABRA3, and the percentage identity and coverage reached 50.15% (Figure 4D). Accordingly, the bHLH transcription factor GLABRA3 was identified as a target transcription factor for binding the promoter region of *PePLDδ* (Figure 4D).

### 2.5. PeGLABRA3 Cloning and Sequence Analysis

**Figure 5:**
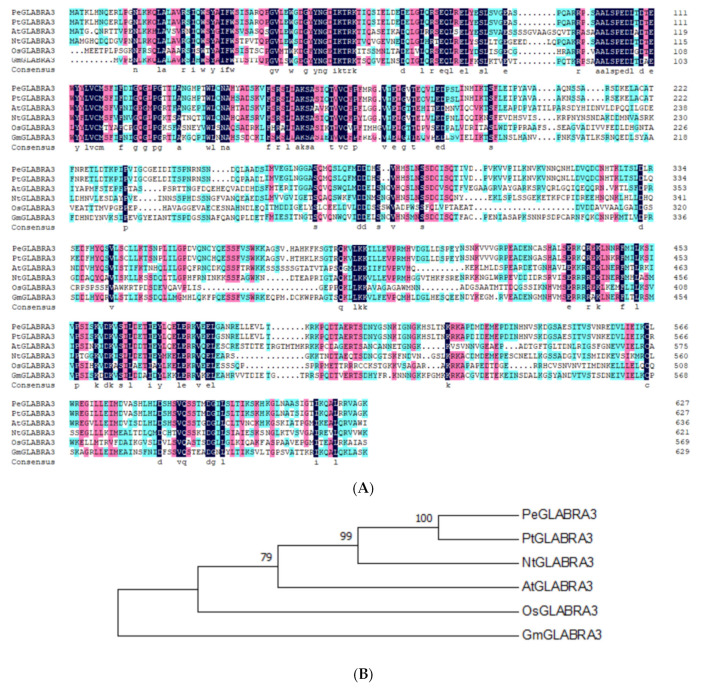
Multiple sequence alignment and phylogenetic analysis of *Populus euphratica* PeGLABRA3. (**A**) Multiple sequence alignment of PeGLABRA3 with GLABRA3 from different species. Black shading indicates identical amino acid residues and blue and pink shadings indicate conserved amino acids, respectively. (**B**) A phylogenetic relationship between PeGLABRA3 and GLABRA3 proteins from other different species. The phylogenetic tree was constructed using the nearest neighbor joining method with MEGA 7.0 software. At, *Arabidopsis thaliana*; Nt, *Nicotiana tabacum*; Pe, *Populus euphratica*; Pt, *Populus trichocarpa*; Os, *Oryza sativa*; Gm, *Glycine max*. GLABRA3 accession numbers for different species are shown in Supplementary Table S2.

The *PeGLABRA3* gene was isolated from the leaves of *P. euphratica* seedlings and used for sequence analysis. A comparison of the amino acid sequences of GLABRA3 from different species revealed that PeGLABRA3 is highly similar to the GLABRA3 of *P. tricocarpa* (Figure 5A). Furthermore, comparative phylogenetic analysis showed that PeGLABRA3 is homologous to tobacco NtGLABRA3 and *P. tricocarpa* PtGLABRA3 (Figure 5B).

### 2.6. NaCl-Induced PeGLABRA3 Expression

*Populus euphratica* increased *PeGLABRA3* transcription in roots and leaves after 1 h of NaCl treatment, and the peak level was observed at 6 h (Figure 1C,D). The pattern of salt-induced *PeGLABRA3* was similar to that of *PePLDδ* during the period of salt stress (Figure 1A–D). Notably, *PeGLABRA3* increased earlier than *PePLDδ* in both roots and leaves (Figure 1A–D).

### 2.7. PeGLABRA3 Binds to the PePLDδ Promoter

To determine whether PeGLABRA3 interacts with *PePLDδ*, the binding of PeGLABRA3 to the *PePLDδ* promoter region was verified by EMSA, Y1H, LRA, and VIGS.

#### 2.7.1. Electrophoretic Mobility Shift Assay

**Figure 6:**
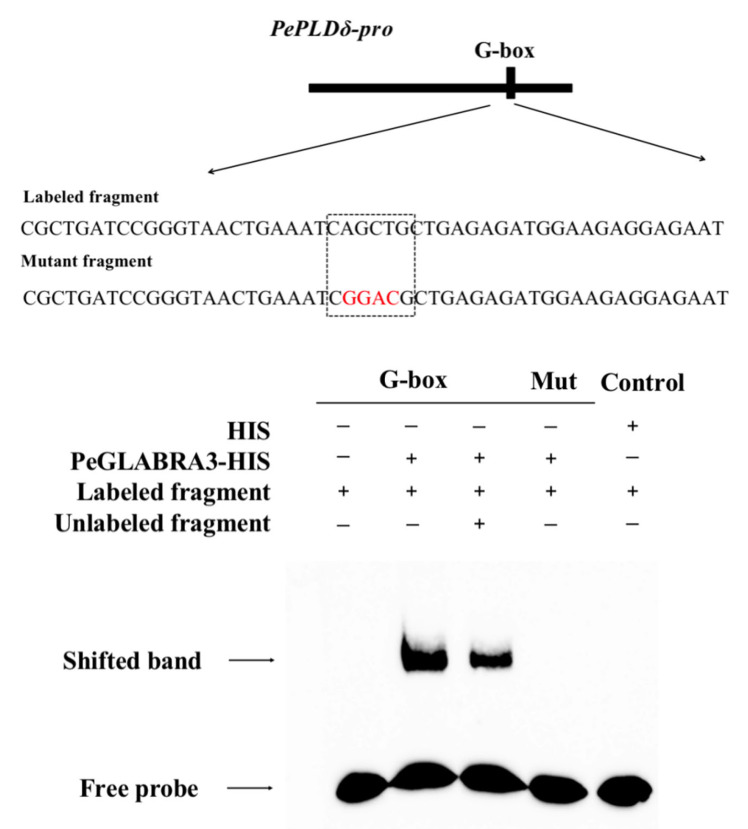
Electrophoretic mobility shift assay (EMSA) verified the interaction of PeGLABRA3 with the *PePLDδ* promoter region. PeGLABRA3-HIS protein purified from prokaryotic expression was used for in vitro EMSA, while HIS-tagged protein was used as a negative control. The mutant probes (Mut, CAGCTG to CGGACG) were used to confirm the binding specificity of G-box to PeGLABRA3. The bases marked in red indicate the mutated bases in the mutant probe. In each loading panel, “+” and “−” indicate the presence or absence of protein and probe, respectively. The cold probe concentration was 10× and the concentration of the polyacrylamide gel was 6%. The EMSA experiment was repeated three times and the representative images are shown.

Previous research showed that the bHLH protein mediates the transactivation of target genes through binding to E- and G-box elements in the promoter region [34,43,44]. The *PePLDδ* promoter region contains a cis-acting element G-box (Figure 2). An EMSA was performed to evaluate the interaction of PeGLABRA3 with the G-box in vitro. Biotin labeling indicated that the PeGLABRA3 protein bound to the G-box motif in the *PePLDδ* promoter, while the negative control, HIS-protein, was unable to bind to the probe (Figure 6, lanes 1, 2, and 5). Furthermore, an increasing ratio of competing probes interfered with the binding of PeGLABRA3 to the biotin-labeled G-box (Figure 6, lane 3). The binding specificity of the G-box to PeGLABRA3 was also confirmed by the mutant G-box. The G-box CAGCTG was mutated to CGGACG and labeled with biotin, and the presence of a G-box mutation probe was found to inhibit the binding of PeGLABRA3 to G-box (Figure 6, lane 4).

#### 2.7.2. Y1H Assay

**Figure 7:**
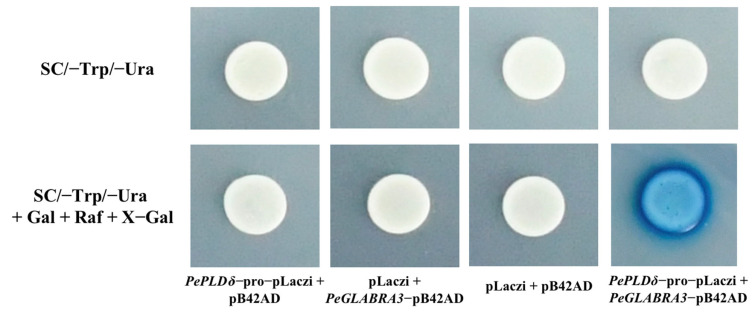
Yeast one-hybrid (Y1H) analysis of PeGLABRA3 binding to the *PePLDδ* promoter. The co-transformation of plasmids for Y1H analysis includes following combinations: (1) *PePLDδ*-pro-pLaczi + pB42AD, (2) pLaczi + *PeGLABRA3*-pB42AD, (3) pB42AD + pLaczi, and (4) *PePLDδ*-pro-pLaczi + *PeGLABRA3*-pB42AD. The yeast cells were cultured on an SD/-Trp/-Ura solid medium supplemented without or with X-Gal. The Y1H analysis was repeated three times and representative images are shown. Gal: galactose, Raf: raffinose.

The 1138 bp promoter sequence of *PePLDδ* was fused to the yeast vector pLaczi, and the full-length coding sequence region of *PeGLABRA3* was fused to the yeast vector pB42AD. Single colonies could be propagated on the two deficient media (SD/-Ura/-Trp), and the blue color of X-Gal was displayed when the vectors carrying *PePLDδ*-pro and *PeGLABRA3* were co-transformed into EGY48 competent cells (Figure 7). In the negative control, color change was not observed (Figure 7). Y1H showed that PeGLABRA3 could interact with the *PePLDδ* promoter.

#### 2.7.3. Luciferase Reporter Assay

**Figure 8:**
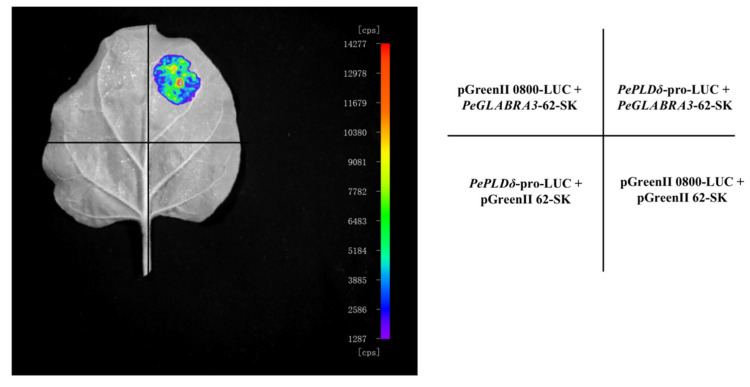
The luciferase reporter assay (LRA) validated the PeGLABRA3 interaction with the *PePLDδ* promoter. *Nicotiana tabacum* leaves were co-transformed with *Agrobacterium* strains containing (1) *PeGLABRA3*-62-SK + pGreenII 0800-LUC, (2) *PeGLABRA3*-62-SK + *PePLDδ*-pro-LUC, (3) *PePLDδ*-pro-LUC + pGreenII 62-SK, or (4) pGreenII 0800-LUC + pGreenII 62-SK. The LRA was repeated three times and representative luciferin luminescence images are shown.

The luminescence of luciferase was extremely low and almost invisible when *A. tumefaciens* vector controls (VCs), pGreenII 0800-LUC, and pGreenII 62-SK were co-transformed into tobacco leaves (Figure 8). The co-transformation of *PePLDδ*-pro-LUC with pGreenII 62-SK and *PeGLABRA3*-62-SK with pGreenII 0800-LUC did not exhibit any visible luminescence (Figure 8). When *PePLDδ*-pro-LUC was co-transformed with *PeGLABRA3*-62-SK into tobacco leaves, a significant increase in luciferase luminescence was observed (Figure 8). An LRA confirmed that PeGLABRA3 activated the *PePLDδ* promoter.

#### 2.7.4. Virus-Induced Gene Silencing

The VIGS was performed in *P. euphratica* seedlings using the tobacco rattle virus (TRV)-based factor pTRV2 [45]. Under NaCl stress, *PeGLABRA3* expression declined correspondingly when *Agrobacterium* carrying TRV2-*PeGLABRA3* was infiltrated into leaves (Figure 1E). It is worth noting that a significant reduction in *PePLDδ* was also observed in TRV-infected leaves (Figure 1F). Accordingly, the silencing of *PeGLABRA3* led to a decrease in *PePLDδ* under salt conditions.

### 2.8. The Overexpression of PeGLABRA3 Enhances Salt Tolerance in Arabidopsis

**Figure 9:**
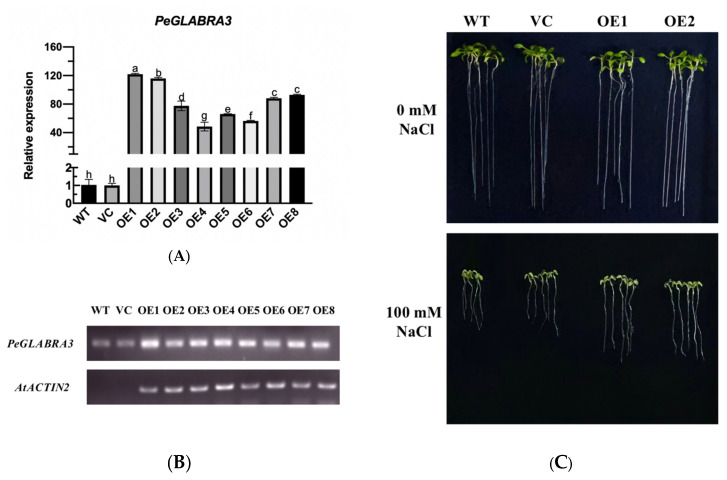

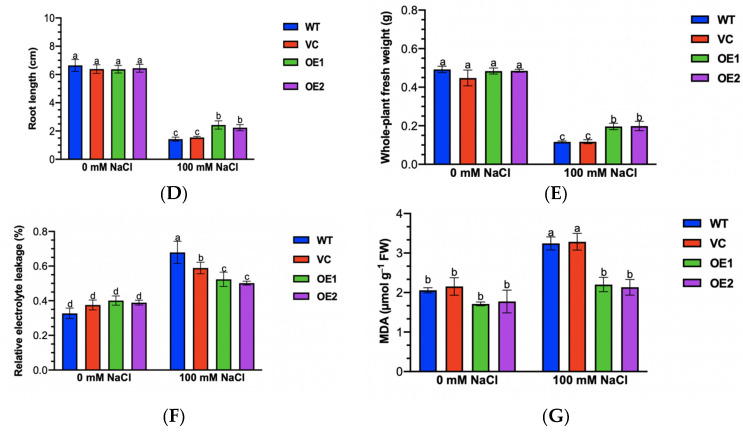
Phenotype tests of *PeGLABRA3*-transgenic lines. (**A***,***B**) RT-qPCR and semiquantitative RT-PCR analysis. Total RNA was isolated from 10-day-old wild type *Arabidopsis thaliana* (WT) seedlings, empty vector control (VC), and transgenic lines (OE1-OE8), and used for semiquantitative PCR and real-time quantitative PCR analysis. Arabidopsis β-actin 2 (*AtACTIN2*) was used as an internal reference gene. Primers designed for *PeGLABRA3* and the internal control gene *AtACTIN2* are shown in Supplementary Table S1. Each column is the mean of three independent experiments, and the bars represent the standard error of the mean. Different letters (a–h) indicate a significant difference at *p* < 0.05. (**C**–**G**) Salt tolerance test. WT and VC seeds and *PeGLABRA3*-transgenic lines OE1 and OE2 were germinated and grown on 1/2 MS medium. Seven-day-old seedlings were exposed to 0 or 100 mM of NaCl for 7 d. Root length, whole-plant fresh weight, relative electrolyte leakage, and MDA content were examined during the period of NaCl treatment. (**C**) Representative pictures showing plant performance and root length under control and NaCl stress. (**D**) Root length. (**E**) Whole-plant fresh weight. (**F**) Relative electrolyte leakage. (**G**) MDA content. Each column is the mean of three independent experiments, and the bars represent the standard error of the mean. Different letters (a–d) indicate a significant difference at *p* < 0.05.

*PeGLABRA3* was overexpressed in Arabidopsis to test whether it could mediate salt tolerance in higher plants. Eight *PeGLABRA3*-transgenic lines, OE1–OE8, were obtained and identified by RT-qPCR and semiquantitative reverse transcription PCR (Figure 9A,B). Two transgenic lines, OE1 and OE2, were used for phenotypic tests due to the higher transcript abundance of *PeGLABRA3* (Figure 9A,B). Compared with WT and VC, root length and whole-plant fresh weight in transgenic lines were less inhibited after NaCl stress (100 mM, 7 d, Figure 9C–E). The salt-increased electrolyte leakage and malondialdehyde (MDA) content were also lower in OE1 and OE2 than in WT and VC (Figure 9F–G).

### 2.9. PeGLABRA3-Transgenic Plants Increased ROS Scavenging Capacity under Salt Stress

**Figure 10:**
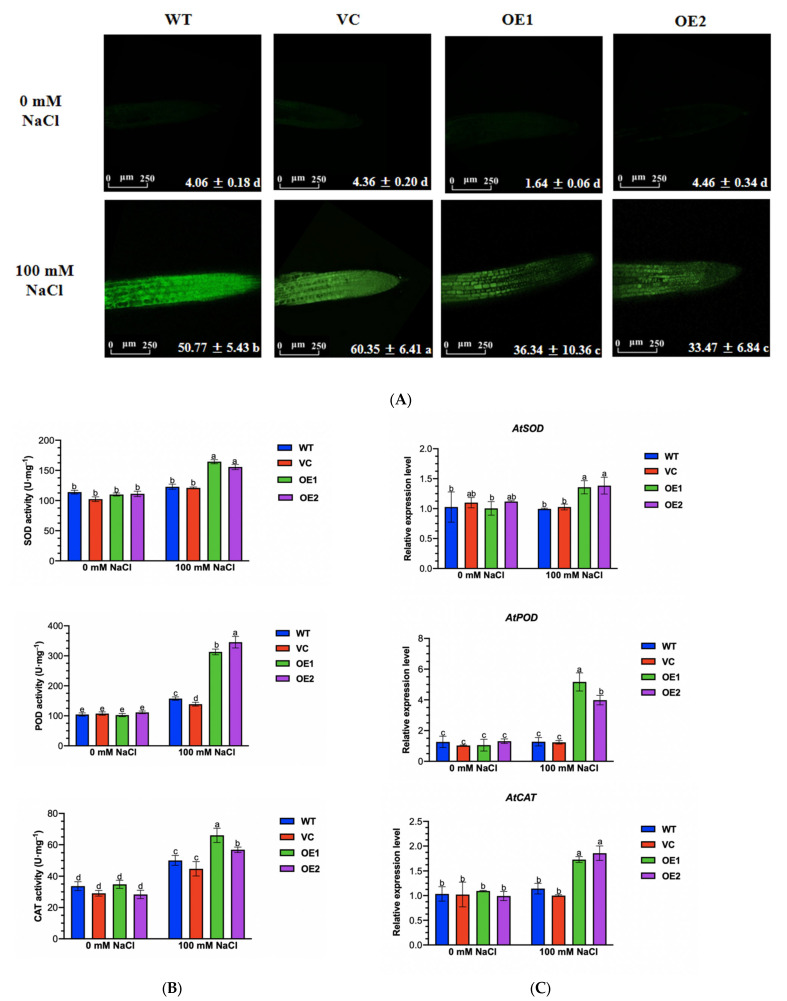
H_2_O_2_ level, activity, and transcription of antioxidant enzymes. (**A**) H_2_O_2_ concentration in root cells. Seven-day-old WT seedlings, VC, and *PeGLABRA3*-transgenic lines OE1 and OE2 were exposed to 0 or 100 mM of NaCl for 12 h. Arabidopsis roots sampled from the control and salt-stressed plants were incubated with 10 μM H_2_DCFDA for 15 min and washed four or five times. The intracellular green fluorescence was detected by a confocal laser microscope. Each value is the mean of three independent experiments, and different letters (a–d) indicate a significant difference at *p* < 0.05. Scale bar = 250 μm. (**B**,**C**) Activity and transcription of antioxidant enzymes. Seven-day-old WT seedlings, VC, and *PeGLABRA3*-transgenic lines OE1 and OE2 were exposed to 0 or 100 mM of NaCl for 7 days. The total activity of SOD, POD, and CAT and transcription of the encoding genes *AtSOD*, *AtPOD*, and *AtCAT* were examined. Arabidopsis β-actin 2 (*AtACTIN2*) was used as the internal reference gene. Primers for *AtSOD*, *AtPOD*, *AtCAT*, and *AtACTIN2* are listed in Supplementary Table S1. Each column is the mean of three independent experiments, and different letters (a–e) indicate a significant difference at *p* < 0.05.

The *PeGLABRA3*-enhanced tolerance to NaCl was associated with increased ROS scavenging and Na^+^ exclusion. H_2_O_2_ concentrations were measured by a fluorescent probe, H_2_DCFDA, in the root cells of all tested genotypes. H_2_DCFDA intensity revealed that WT and VC had 1.38- to 1.81-fold higher H_2_O_2_ than the *PeGLABRA3*-transgenic lines OE1 and OE2 after NaCl treatment (100 mM, 12 h, Figure 10A). The increased ability to control ROS in transgenic lines was related to the enhanced activity of SOD, POD, and CAT and the increased transcription of the genes encoding these antioxidant enzymes. In *PeGLABRA3*-transgenic lines, the total enzyme activities of SOD, POD, and CAT increased by 10–54% under salt treatment, which was significantly higher than in WT and VC (Figure 10B). The salt-induced transcription of *SOD*, *POD*, and *CAT* showed a trend similar to that of antioxidant enzymes (Figure 10C).

### 2.10. PeGLABRA3-Transgenic Plants Maintained Ionic Homeostasis under Salinity

**Figure 11:**
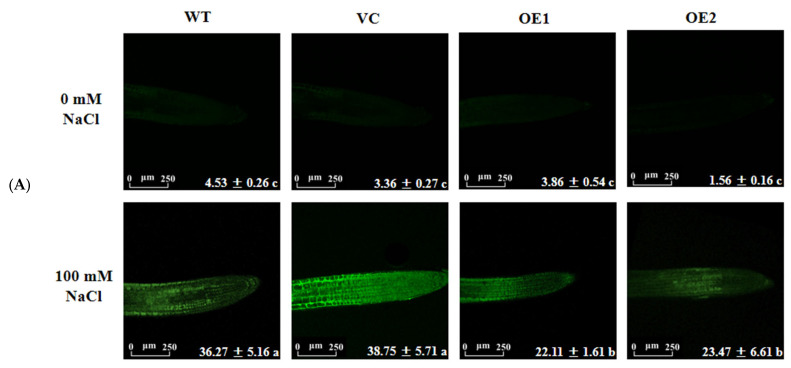

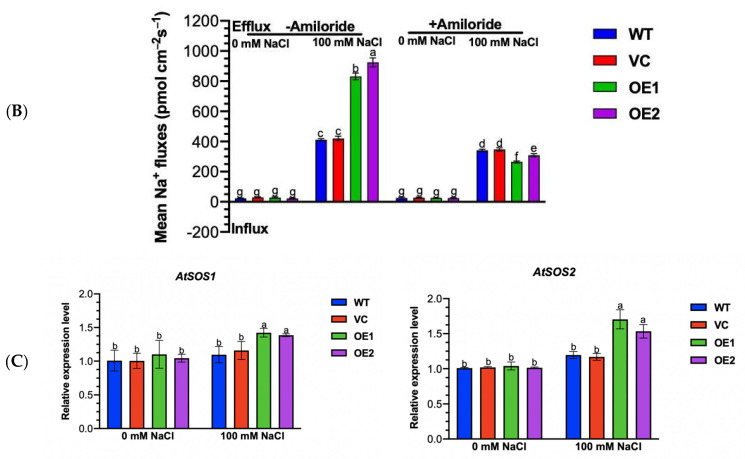
Na^+^ concentration and Na^+^ flux in the root. (**A**) Na^+^ concentration in root cells. Seven-day-old WT seedlings, VC, and *PeGLABRA3*-transgenic lines OE1 and OE2 were exposed to 0 or 100 mM of NaCl for 12 h. Arabidopsis roots sampled from control and salt-stressed plants were incubated with 20 μM CoroNa™Green for 2 h and washed four or five times. The intracellular green fluorescence was detected by a confocal laser microscope. Each value is the mean of three independent experiments and different letters (a–c) indicate a significant difference at *p* < 0.05. Scale bar = 250 μm. (**B**) Na^+^ flux in the root tip. Seven-day-old WT seedlings, VC, and *PeGLABRA3*-transgenic lines OE1 and OE2 were exposed to 0 or 100 mM of NaCl for 12 h. Then, Arabidopsis roots were incubated with amiloride (an inhibitor of Na^+^/H^+^ antiporter, 0 or 5 mM) for 30 min. Thereafter, roots were sampled and equilibrated for 30 min in the basic solution containing 0.1 mM of NaCl, 0.1 mM of CaCl_2_, 0.1 mM of MgCl_2_, 0.5 mM of KCl, and 2.5% sucrose (pH 5.8). Na^+^ fluxes were monitored by NMT microelectrodes at the meristematic region (200 µm from the root tip) and continuously recorded for 10 min. Each column is the mean of three independent experiments, and different letters (a–g) indicate a significant difference at *p* < 0.05. (**C**) Transcription of *AtSOS1* and *AtSOS2*. Seven−day−old WT seedlings, VC, and *PeGLABRA3*-transgenic lines OE1 and OE2 were exposed to 0 or 100 mM of NaCl for 7 d. Transcription of *AtSOS1* and *AtSOS2* was examined, and Arabidopsis β-actin 2 (*AtACTIN2*) was used as the internal reference gene. Primers for *AtSOS1*, *AtSOS2*, and *AtACTIN2* are listed in Supplementary Table S1. Each column is the mean of three independent experiments, and different letters (a and b) indicate a significant difference at *p* < 0.05.

The Na^+^-specific probe CoroNa^TM^Green was used to determine Na^+^ content in root cells. The fluorescence intensity significantly increased in root cells of all tested lines upon salt exposure (NaCl 100 mM, 12 h, Figure 11A). It was noteworthy that the cellular Na^+^ was significantly lower in transgenic lines than in WT or VC under salinity (Figure 11A). To determine whether the low Na^+^ accumulation in *PeGLABRA3*-transgenic plants was the result of an active salt extrusion, a noninvasive micro-test technique (NMT) was used to detect Na^+^ flow in the roots. The short-term NaCl treatment (100 mM, 12 h) resulted in a marked rise of Na^+^ efflux in the root tips of all tested lines (Figure 11B). We noticed that *PeGLABRA3*-transgenic lines exhibited a 2-fold higher flux rate than WT and VC (Figure 11B). However, the addition of amiloride, an inhibitor of Na^+^/H^+^ anti-transport, reduced the salt-elicited Na^+^ efflux in WT, VC, and *PeGLABRA3*-transgenic lines (Figure 11B), suggesting that the Na^+^ efflux was the result of Na^+^/H^+^ antiport across the PM. Here, we also examined the abundance of salt overly sensitive (SOS) pathway genes, *SOS1* and *SOS2*, in Arabidopsis plants. The transcription of *AtSOS1* and *AtSOS2* was significantly upregulated by NaCl in *PeGLABRA3*-transgenic lines (Figure 11C). This finding indicates that the Na^+^ extrusion resulted from the Na^+^/H^+^ antiport in the roots of *PeGLABRA3*-transgenic plants.

### 2.11. PeGLABRA3 Enhances AtPLDδ Transcription by Binding Its Promoter

**Figure 12:**
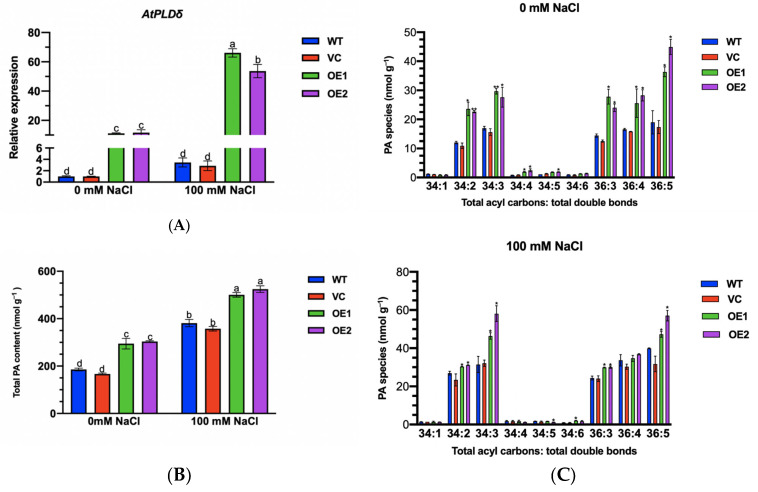
*AtPLDδ* transcription and phosphatidic acid (PA) content. (**A**) *AtPLDδ* transcription. WT seedlings, VC, and *PeGLABRA3*-transgenic lines OE1 and OE2 were germinated and grown on 1/2 MS medium for 7 d. The seedlings were transferred to a liquid medium containing 0 or 100 mM of NaCl for a further 7 d. Transcription of *AtPLDδ* was examined, and Arabidopsis β-actin 2 (*AtACTIN2*) was used as the internal reference gene. Primers for *AtPLDδ* and *AtACTIN2* are listed in Supplementary Table S1. Each column is the mean of three independent experiments, and different letters (a–d) indicate a significant difference at *p* < 0.05. (**B***,***C**) Phosphatidic acid (PA) content. Seven-day-old WT seedlings, VC, and *PeGLABRA3*-transgenic lines were transferred to a liquid medium containing 0 or 100 mM of NaCl for 24 h. Seedlings sampled from control and stressed plants were used to measure total PA content and the concentration of specific PA species, including 34:1, 34:2, 34:3, 34:4, 34:5, 34:6, 36:3, 36:4, and 36:5. Phosphatidic acid species were identified and quantified using electrospray ionization tandem mass spectrometry. Each column is the mean of three independent plants, and different letters (a–d) or asterisks (*) represent significant differences at *p* < 0.05, (**) represent significant differences at *p* < 0.01.

The *PeGLABRA3*-transgenic lines OE1 and OE2 typically had higher expression of *AtPLDδ* than WT and VC in the presence and absence of salt treatment (Figure 12A). To explore the transcriptional regulation of *AtPLDδ*, the promoter region of *AtPLDδ* was cloned and analyzed (Figure S1). The cis-element G-boxes were shown to be in the promoter region (Figure S1). Electrophoretic mobility transfer analysis and luciferase reporter gene assay were performed to determine whether PeGLABRA3 activated *AtPLDδ* transcription in transgenic plants. EMSA and LRA data revealed that PeGLABRA3 binds to the *AtPLDδ* promoter region (Figures S2 and S3). Therefore, PeGLABRA3 interacted with the *AtPLDδ* promoter to accelerate *AtPLDδ* transcription.

### 2.12. Phosphatidic Acid Content of PeGLABRA3-Transgenic Lines

Membrane phospholipids can be hydrolyzed by phospholipase Dδ to produce phospholipid acid [20] and, therefore, we determined the content of PA in transgenic lines. NaCl increased the content of total PA in all tested lines, but *PeGLABRA3*-transgenic plants retained 1.25- to 1.50-fold higher PA than WT and VC irrespective of control and salt treatment (Figure 12B). Moreover, the major PA species were further analyzed in Arabidopsis. Compared with WT and VC, the abundance of typical PA species in *PeGLABRA3*-transgenic lines increased, including PA species 34:2(16:0–18:2), 34:3(16:0–18:3), 36:3(18:1–18:2), 36:4(18:1–18:3), and 36:5(18:2–18:3) (Figure 12C). Notably, the PA species 34:2(16:0–18:2), 34:3(16:0–18:3), and 36:5(18:2–18:3) significantly increased in transgenic lines after NaCl exposure (Figure 12C).

## 3. Discussion

### 3.1. PeGLABRA3 Interacts with PePLDδ to Increase Gene Expression under NaCl Stress

NaCl induced the expression of *PeGLABRA3*, a bHLH transcription factor, in the roots and leaves of *P. euphratica* (Figure 1). Similarly, NaCl was found to upregulate a number of *bHLH* genes in Arabidopsis [34], rice [35], and sugar beet [36]. Previously, bHLH transcription factors were shown to bind to the E-box and GCG-box motifs to regulate the expression of genes involved in salt stress tolerance [34,43,44]. *PePLDδ* was found to contain G-box in the promoter region (Figure 2) and became active in roots and shoots after NaCl exposure (Figure 3). By means of DNA pull-down and mass spectrometry, Y1H, LRA, and EMSA, we confirmed that PeGLABRA3 binds the *PePLDδ* promoter (Figure 4, Figure 6, Figure 7 and Figure 8). Furthermore, *PePLDδ* expression was downregulated when the *PeGLABRA3* gene was silenced in salt-treated *P. euphratica* leaves (Figure 1). Therefore, the salt-related increase in the expression of *PePLDδ* was due to the binding of PeGLABRA3 to the promoter of *PePLDδ*. We have previously shown that *P. euphratica* phospholipase Dδ increases salt tolerance by regulating K^+^/Na^+^ and ROS homeostasis in Arabidopsis [20]. Here, the binding of PeGLABRA3 to the *AtPLDδ* promoter was able to improve salt stress in transgenic Arabidopsis.

### 3.2. PeGLABRA3 Interacts with AtPLDδ to Mediate Ionic and ROS Homeostasis

The *PeGLABRA3*-transgenic lines showed enhanced root length and whole-plant growth under salinity (Figure 9). This outcome accords with previous findings that the overexpression of various *bHLH*s, such as *AtbHLH92* [37], *AtbHLH112* [34], *OrbHLH2* [46], *BvBHLH93* [36], *VvbHLH1* [38], and *MxbHLH18* [39], enhances salt tolerance in transgenic plants. In this study, PeGLABRA3 enhanced salt tolerance through an increased ability to control ionic and ROS homeostasis in transgenic Arabidopsis. *PeGLABRA3*-transgenic lines increased the activities of antioxidant enzymes such as SOD, POD, and CAT by positively regulating the expression of antioxidant genes (Figure 10). The upregulated ROS scavenging enzymes enabled the salt-stressed plants to maintain a low level of H_2_O_2_ (Figure 10). As a result, salt-induced oxidative damage was reduced (Figure 9). Similarly, the overexpression of *bHLH*s, including *BvBHLH93* [36], *VvbHLH1* [38], *MxbHLH18* [39], and *NtbHLH123* [40], can enhance the activities of antioxidant enzymes and the expression of antioxidant genes. Tobacco transcription factor *bHLH123* improves salt tolerance by activating NADPH oxidase *NtRbohE* expression, which mediates the rapid accumulation of ROS and the response to salt stress during the early stages [40]. Our data showed that PeGLABRA3 activated *AtPLDδ* transcription to maintain ROS homeostasis under salt stress. The Arabidopsis *AtPLDδ* contains several G-boxes in the promoter region (Figure S1). The EMSA and LRA demonstrated that PeGLABRA3 binds to the *AtPLDδ* promoter region (Figures S2 and S3). In accordance with those results, the expression of *AtPLDδ* was significantly higher in transgenic lines than in WT and VC under control conditions and upon salt exposure (Figure 12). The PeGLABRA3-activated *AtPLDδ* resulted in increased PA in transgenic lines, including PA species such as 34:2 (16:0–18:2), 34:3 (16:0–18:3), 36:3 (18:1–18:2), 36:4 (18:1–18:3), and 36:5 (18:2–18:3) in no-salt controls and 34:2 (16:0–18:2), 34:3 (16:0–18:3), and 36:5 (18:2–18:3) in salt-stressed plants (Figure 12). Previous studies showed that Arabidopsis overexpressing *P. euphratica PePLDδ* had increased PA species and upregulated antioxidant enzymes SOD, POD, and APX [20]. Therefore, PeGLABRA3 interacted with the promoter of *AtPLDδ* to enhance gene expression, and the AtPLDδ-derived PA contributed to enhancing antioxidant defense under salt stress. In accordance, the overexpression of Arabidopsis *AtPLDα* in poplars increases the activity of SOD, CAT, and POD under drought treatment [16]. The AtPLDα-mediated antioxidant enzymes are essential for Arabidopsis to adapt to osmotic stress [47]. Furthermore, Arabidopsis phospholipase Dδ is involved in the regulation of ROS-mediated microtubule dynamic organization, stomatal movement, and heat tolerance [48].

PeGLABRA3 also helped transgenic plants maintain ionic homeostasis under salt stress. *PeGLABRA3*-transgenic lines showed a greater capacity for Na^+^ extrusion under salinity (Figure 11). Similarly, the *BvbHLH93* expression lines accumulated less Na^+^ but more K^+^ than the WT [36]. bHLH transcription factors have been shown to confer K^+^/Na^+^ homeostasis by mediating the expression of *KUP2* [41], *NHX1*, and *NHX6* [42] in Arabidopsis. In rice, bHLH transcription factors regulate the Na^+^/K^+^ ratio in salt stress by increasing the expression of *OsHKT1s* [35]. Here, PeGLABRA3 interacted with *AtPLDδ* to increase PA levels, contributing to Na^+^ homeostasis as *PePLDδ* overexpression increased Na^+^ extrusion in Arabidopsis [20]. It was previously demonstrated that NaCl-induced PA interacts with mitogen-activated protein kinase 6 (MPK6), which directly phosphorylates the downstream Na^+^/H^+^ antiporter, *SOS1* [49]. Here, *PeGLABRA3*-transgenic lines exhibited increased levels of *AtSOS1* and *AtSOS2*, indicating that PeGLABRA3 interacts with *AtPLDδ* to produce specific PA species, such as 34:2 (16:0–18:2 PA), which activated SOS1 for Na^+^ extrusion (Figure 11) [49].

## 4. Materials and Methods

### 4.1. Plant Material and Salt Treatment

*Populus euphratica* seedlings, obtained from Xinjiang Uygur Autonomous Region, were planted in 10 L pots containing a 1:1 ratio (*v*/*v*) of sand and soil. After 3 months of culture in a greenhouse at Beijing Forestry University, *P. euphratica* seedlings were exposed to 200 mM of NaCl for 48 h [45]. Roots and mature leaves were sampled after 0, 1, 3, 6, 12, 24, and 48 h of salt treatment and used for RT-qPCR analysis.

### 4.2. RNA Isolation and Full-Length Cloning of PeGLABRA3

Using the EASYspin Plus Complex Plant RNA Kit (Aidlab Biotech, Beijing, China), the total RNA was extracted from *P. euphratica* leaves, according to the manufacturer’s instructions. The first-strand cDNA was synthesized through a reverse transcription reaction. The forward and reverse primers were designed based on the homologous sequences of *P. euphratica GLABRA3*. The 5′-3′ primer sequence was as follows: forward, 5′ -ATGGCTACTAAGCTCCACAACC-3′, and reverse, 5′-TCAACATTTCCCAGCAACTCTTC-3′. The PCR products were gel-purified and ligated into a pMD18-T vector (Takara, Dalian, China) for DNA sequencing.

### 4.3. PeGLABRA3 Sequence and Phylogenetic Analysis

We used the ClustalW (http://www.genome.jp/tools/clustalw/, (accessed on 17 January 2021) EMBL-EBI, Hinxton, Cambridgeshire, UK) for the multiple sequence alignments of GLABRA3 proteins. A phylogenetic tree was constructed by MEGA 5.2 software (http://www.megasoftware.net/index.php (accessed on 17 January 2021), Center for Evolutionary Medicine and Informatics, Tempe, AZ, USA). The GLABRA3 homologs used for multiple sequence alignment and phylogenetic analysis are listed in Supplementary Table S2.

### 4.4. DNA Pull-Down

#### 4.4.1. Probe Design and Labeling

Upper mature leaves were sampled from *P. euphratica* seedlings and used to isolate genomic DNA and total protein [45]. Using genomic DNA as the template, the *PePLDδ* promoter fragment was amplified by PCR, cloned, and sequenced. The probes were biotin-labeled and purified with a gel extraction kit (Sigma-Aldrich, St. Louis, MI, USA). The concentration of the probes was detected, and they were stored at −20 °C for DNA pull-down.

#### 4.4.2. DNA Pull-Down and Mass Spectrometry Analysis

The biotin-labeled DNA and nucleic acid were buffered into a 500 µL reaction system, to which magnetic beads (Beaver Bio 22305-1) were added. The system was incubated at room temperature for 1 h to form a DNA-magnetic bead complex. The supernatant was removed from the magnetic rack for subsequent bead hanging efficiency testing. The DNA-magnetic bead complex was further washed with precooled nucleic acid incubation buffer (50 mM Tris-HCl, pH 7.6, 0.1 mM EDTA, 0.1% Tween 20) and with a protein incubation buffer (50 mM Tris-HCl, pH 7.6, 0.1 mM EDTA, 0.1% Tween 20, 75 mM KCl, 1 mM MgCl_2_, 10% glycerol). The washing was repeated twice prior to magnetic separation on a magnetic frame. Protein extracts of *P. euphratica* leaves were diluted to 500 µL with a protein extraction buffer and incubated with the DNA-magnetic bead complex overnight at 4 °C to form a protein-DNA-magnetic bead complex. The supernatant was removed completely after magnetic separation on a magnetic frame. Then, the magnetic beads were rinsed with a cold protein incubation buffer six or seven times to collect protein. The protein sample was diluted with 100 µL of a protein elution buffer, bathed at 95 °C for 5 min, and centrifuged at 12,000 rpm for 5 min. The enriched protein was separated via SDS-PAGE and stained with a silver staining kit (Sigma-Aldrich). Finally, the obtained proteins were analyzed with mass spectrometry (Ultraflex III mass spectrometer, Bruker, Germany). Primary mass spectra were scanned at a 250 ms ion accumulation time, and secondary mass spectra of 30 precursor ions were collected at a 50 ms ion accumulation time. The MS1 spectrum was collected in the range of 350–1200 m/z, and the MS2 spectrum was collected in the range of 100–1500 m/z. The dynamic exclusion time of precursor ions was set to 15s.

The database used for protein determination was Uniprot_*Arabidopsis thaliana* (https://www.uniprot.org/uniprotkb?query=Arabidopsis%20 thaliana, accessed on 13 August 2020), Uniprot_*Populus euphratica* (https://www.uniprot.org/uniprotkb?query=Populus%20euphratica, accessed on 16 December 2020), and Uniprot_*Populus trichocarpa* (https://www.uniprot.org/uniprotkb?query=Populus%20trichocarpa, accessed on 16 December 2020). The proteins identified by DNA pull-down and mass spectrometry assay are shown in Supplementary Table S3.

### 4.5. Promoter Cloning and Sequence Analysis

The promoter of *PePLDδ,* the 1138 bp promoter fragment upstream of the translation initiation codon (−1138 to 0 bp), was isolated from *P. euphratica* leaves via PCR amplification [45]. The isolated 1138 bp region corresponds to the intergenic region between the *PePLDδ* gene and its upstream gene. The site of *PePLDδ* is 2,467,415–2,474,267, and its upstream gene is 2,456,149–2,463,149. The *PePLDδ-*pro (1138 bp) sequence was analyzed using the PLACE and PlantCARE databases. The promoter of *A. thaliana AtPLDδ* was also cloned and analyzed, as described above. The primers used for the isolation of *P. euphratica PePLDδ* and *A. thaliana AtPLDδ* are shown in Supplementary Table S1. The 1237 bp DNA sequence of the *AtPLDδ* promoter contained the following cis-acting elements: ARE (a cis-acting regulatory element essential for the anaerobic induction), ABRE motif (a cis-acting element involved in the abscisic acid responsiveness), MYC (a cis-acting element in response to drought and ABA), and G-box (a cis-regulatory element involved in light response). ATG was the start codon of the *AtPLDδ* gene (Supplementary Figure S1).

### 4.6. Luciferase Reporter Assay

LRA was performed following Yao et al. (2020) [45] and Hellens et al. (2005) [50]. In brief, the coding sequence region of the *PeGLABRA3* gene was cloned and constructed into the transient expression vector pGreenII 62-SK. The promoter sequence of *PePLDδ* was cloned into the LUC reporter gene of the pGreenII 0800-LUC vector. After vector construction, the recombinant plasmid was transformed into the competent state of *Agrobacterium tumefaciens* GV3101 (pSOUP-P19). According to the transformation steps, 100 μL of incubated bacterial liquid was finally absorbed and coated on an LB solid medium containing 50 μg/mL kanamycin and 25 μg/mL rifampicin, and the culture was inverted at 28 °C for 48 h. Positive clones identified by PCR were selected and incubated at 28 °C in a shaker (220 rpm) until optical density (OD_600_) reached 1.0. The bacteria were collected by centrifugation at 4000 rpm for 5 min at room temperature; thereafter, OD_600_ was diluted to 0.6 with tobacco injection buffer. The bacterial solution containing the plasmid *PePLDδ-*pro-LUC or pGreenII 0800-LUC was mixed with the same volume of a bacterial solution containing the plasmid *PeGLABRA3*-62-SK or pGreenII 62-SK, left at room temperature for 2 to 4 h, and then injected into tobacco leaves using a 1 mL syringe. After 48–60 h of dark treatment, D-luciferin (1 mM) was coated on the leaves, and the LB983 Night Owl II Living molecular Imaging system (Berthold Technologies, Bad Wildbad, Germany) was used to observe the fluorescence. The LRA was repeated at least three times.

The LRA was also used to validate the PeGLABRA3 interaction with the *A. thaliana AtPLDδ* promoter, as mentioned above.

### 4.7. Yeast One-Hybrid Assay

The promoter of *PePLDδ* (1138 bp) containing the G-box was cloned and ligated into the pLaczi vector. PeGLABRA3 was cloned and expressed by fusion with the pB42AD vector. The linearized *PePLDδ*-pro-pLaczi plasmid was co-transformed with pB42AD or *PeGLABRA3*-pB42AD into Y1H competent cells (EGY48), and the *PeGLABRA3*-pB42AD plasmid was co-transformed with pLaczi or *PePLDδ*-pro-pLaczi into Y1H yeast. The mixes were incubated at 30 °C for 30 min and resuspended every 15 min, followed by incubation at 42 °C for 15 min with resuspension every 7.5 min. The Y1H yeast cells collected by centrifugation were evenly cultured on an SD/-Trp/-Ura solid medium and incubated at 30 °C for 2–4 days. After growing single colonies, the cells were spread and coated on an SD/-Trp/-Ura + Gal + Raf + X-Gal color plate for observation.

### 4.8. Electrophoretic Mobility Shift Assay

The full-length coding sequence of *PeGLABRA3* was cloned with HIS-tag at the C-terminus of the pET28a vector. An empty pET28a vector was used as a negative control. The constructed *PeGLABRA3*-pET28a vector was transferred to *Escherichia coli* competent cells (BL21) for re-transformation. The cells were incubated in LB medium at 37 °C until OD_600_ reached 0.4 to 0.6 and were then incubated at 16 °C for 12–16 h after induction with 0.5 mM isopropyl β-D-1-thiogalactopyranoside. The bacteria were collected by centrifugation, resuspended with an 8 mL HIS protein resuspension buffer, and then disrupted by ultrasound for 15 min before centrifugation. The supernatant was added to a packed column that had already been washed 15 times with the volume of the resuspended buffer. After 2 h of incubation on ice, the resuspended buffer was allowed to flow out from the bottom. The column was then washed four or five times with the resuspended buffer containing 50 mM imidazole (5×), followed by two or three washes with the gradient buffer containing 100 mM and 150 mM imidazole. Finally, 0.5 mL of an elution buffer containing 400 mM imidazole was added to the packed column, and samples were collected in centrifuge tubes. The protein collection was repeated three or four times, and the samples were stored for later use.

For biotin labeling, the 50 bp DNA fragment of the *PePLDδ* promoter,5′-CGCTGATCCGGGTAACTGAAATCAGCTGCTGAGAGATGGAAGAGGAGAAT-3′ and 5′-ATTCTCCTCTTCCATCTCTCAGCAGCTGATTTCAGTTACCCGGATCAGCG-3′ (harboring the G-box base sequence) were biotin-labeled at the 3′ ends using an EMSA Probe Biotin Labeling Kit (Beyotime, Nantong, China). A cold competition experiment was conducted with probes that were not labeled. Biotin-labeled mutational probes were used to verify the specificity of the PeGLABRA3 binding to G-boxes (CAGCTG). The mutant probes (Mut, CAGCTG to CGGACG) were used to confirm the binding specificity of the G-box to PeGLABRA3. Representative results were obtained by repeating the EMSA three times.

The EMSA verification of the PeGLABRA3 binding to the *AtPLDδ* promoter was performed as described above. The 50 bp DNA fragment of the *AtPLDδ* promoter used for biotin labeling were 5′-AACTCCCATCACGTCGTCCCTCCACCTGTCCTCTCTTCTC CTTCCTTGCT-3′ and 5′-AGCAAGGAAGGAGAAGAGAGGACAGGTGGAGGGAC GACGTGATGGGAGTT-3′ (harboring the G-box base sequence), respectively. The mutant probes (Mut, CACCTG to CTGGTG) were used to confirm the binding specificity of the G-box to PeGLABRA3.

### 4.9. Construction and Transformation of the PePLDδ-pro::GUS Gene

The *PePLDδ* promoter was cloned into the pBI121 vector. Then, *PePLDδ*-pro was combined with *β-glucuronidase* gene (GUS) and transformed into *Agrobacterium tumefaciens*. PBI121-*PePLDδ*-pro::GUS was transferred into wild type Arabidopsis. Transgenic lines were selected on 1/2 MS medium containing kanamycin resistance. WT seedlings and *PePLDδ*-Pro::GUS transgenic lines were germinated in 1/2 MS medium. The seedlings were treated with 0 or 100 mM of NaCl for 24 h after 10 days of culture. Roots and leaves were sampled for GUS staining [45]. The GUS staining was repeated with three to five individual plants for each treatment.

### 4.10. Virus-Induced Gene Silencing of PeGLABRA3 in P. euphratica

VIGS, a technique that leads to specific degradation of the mRNA of the target gene, was carried out as previously described [51,52]. Briefly, a 250 bp length of the *PeGLABRA3* gene was obtained with two specific primers: F: 5′-GGTACCATGGCTACTAAGCTCCACAA-3′ and R: 5′-GAGCTCTCAACATTTCCCAGCAACTC-3′. The *PeGLABRA3* fragment was reassembled to the pTRV2 expression vector. Then, the pTRV2-*PeGLABRA3* and pTRV1 plasmids were transformed into *Agrobacterium* GV3101, respectively. *Agrobacterium tumefaciens* containing pTRV2-*PeGLABRA3* and pTRV1 was mixed at a ratio of 1:1 and injected into the leaves of *P. euphratica* seedlings. After 45 days of VIGS, *P. euphratica* was subjected to 200 mM of NaCl for 24 h. Then, upper mature leaves were collected for RT-qPCR analysis.

### 4.11. Generation of PeGLABRA3-Transgenic Lines

Wild type Arabidopsis seeds were sown in a 1/2 MS solid medium, vernalized at 4 °C for 3 d, and cultured in a climate chamber. The temperature was maintained at 22 °C, and humidity was maintained at 60%. The long day photoperiod was 16 h light/8 h dark. After 10 d of culture, the germinated seedlings with four cotyledons were transferred to the nursery soil mixed with vermiculite at a ratio of 1:1 and cultured in the climate chamber.

The full-length *PeGLABRA3* gene was inserted into the expression vector pCAMBIA1300-GFP containing the promoter of *CaMV35S*. The recombinant plasmid pCAMBIA1300-GFP-*PeGLABRA3* was then transformed into *A. tumefaciens*. The empty vector pCAMBIA1300-GFP was introduced into WT Arabidopsis plants as a control. Homozygous seeds were screened in a solid medium containing 25 mg/L hygromycin. The expression level of *PeGLABRA3* in T3 homozygous transgenic lines was quantified by semiquantitative reverse transcription PCR and RT-qPCR.

### 4.12. Phenotype Tests of Transgenic Lines

Wild type *Arabidopsis thaliana* (WT) seedlings, empty vector control (VC), and *PeGLABRA3*-transgenic lines (OE1 and OE2) were sterilized with 1% sodium hypochlorite for 10 min, washed five or six times, and grown in 1/2 MS medium. Uniform seedlings (seven-day-old) of all tested lines were placed vertically in a solid 1/2 MS medium supplemented with 0 or 100 mM of NaCl for another seven days of salt exposure. Thereafter, the root length and fresh weight were measured. The root length was examined using ImageJ Pro6 (https://imagej.net/ij/index.html (accessed on 27 July 2021)). The salt tolerance test of WT, VC, and transgenic lines was repeated at least three times.

Mature leaves were sampled from WT, VC, and *PeGLABRA3*-transgenic lines treated with or without 100 mM of NaCl. Relative electrolyte leakage and MDA content were determined as previously described [20].

### 4.13. Na^+^ Flux Measurements

Seven-day-old WT seedlings, VC, and *PeGLABRA3*-transgenic lines OE1 and OE2 were treated with 0 or 100 mM of NaCl for 12 h. Then, Arabidopsis roots were incubated with amiloride (an inhibitor of Na^+^/H^+^ antiporter, 0 or 5 mM) for 30 min. Roots were sampled and equilibrated for 30 min in the basic solution containing 0.1 mM of NaCl, 0.1 mM of CaCl_2_, 0.1 mM of MgCl_2_, 0.5 mM of KCl, and 2.5% sucrose (pH 5.8). Root tips were sampled for Na^+^ flux recordings with the noninvasive micro-test technique (NMT-YG-100, Younger USA, LLC, Amherst, MA, USA). Na^+^ fluxes were monitored by NMT microelectrodes at the meristematic region (200 µM from the root tip) and continuously recorded for 10 min [53]. Five or six individual plants were examined for each treatment. 

### 4.14. Cellular Na^+^ and H_2_O_2_ Determination Roots

The fluorescent probe CoroNa^TM^Green was used to detect Na^+^ content in control and short-term NaCl-stressed roots (0 or 100 mM NaCl, 12 h). Roots were immersed in 5 mM of an MES-KCl loading buffer (pH 5.7) containing CoroNa^TM^Green (20 µM), incubated at room temperature for 2 h in the dark, and then rinsed four to five times with MS solution. For H_2_O_2_ determination, Arabidopsis roots were cultured in 10 μM H_2_DCFDA (Molecular Probe, Eugene, OR, USA) for 15 min and washed four or five times. The fluorescence of CoroNa^TM^Green and H_2_DCFDA was measured using a Leica SP8 confocal microscope at an excitation wavelength of 488 nm and an emission wavelength of 510 to 530 nm. Relative fluorescence intensity was calculated by Image Pro Plus 6.0 (Media Cybernetics, Silver Spring, MD, USA) [45].

### 4.15. Determination of Activity and Transcription of Antioxidant Enzymes

Seven-day-old WT seedlings, VC, and *PeGLABRA3*-transgenic lines OE1 and OE2 were exposed to 0 or 100 mM of NaCl for 7 days. Control and salt-stressed plants were sampled to measure the activities of SOD, POD, and CAT [20]. The transcription of *AtSOD*, *AtPOD*, and *AtCAT* in the control and salinized plants was also examined with RT-qPCR [20].

### 4.16. RT-qPCR Analysis

Total RNA was extracted from *P. euphratica* roots, leaves, and WT Arabidopsis seedlings, VC, and *PeGLABRA3*-overexpressed lines using the EASYspin Plus Complex Plant RNA Kit (Aidlab Biotech, Beijing, China) [45]. The templates for RT-qPCR were prepared by reverse transcription, as previously described [20,54]. *PeACT7* and *AtACTIN2* were used as the internal reference genes for *P. euphratica* and Arabidopsis, respectively. Forward and reverse primers designed to target *PeGLABRA3*, *PePLDδ*, *AtSOS1*, *AtSOS2*, *AtSOD*, *AtPOD*, *AtCAT*, *PeACT7*, and *AtACTIN2* are listed in Supplementary Table S1. Three biological samples were set for each treatment.

### 4.17. Quantitative Analysis of PA Species

Seven-day-old WT seedlings, VC, and *PeGLABRA3*-transgenic lines (OE1 and OE2) were subjected to 0 or 100 mM of NaCl for 24 h in a liquid MS medium. Electrospray ionization tandem mass spectrometry was used to analyze and quantify the lipids after treatment [20,55].

### 4.18. Data Analysis

The experimental data were subjected to SPSS version 19.0 (IBM Corporation, Armonk, NY, USA) for normality and homogeneity of variances tests. Significant differences between means were determined by Duncan’s multiple range test. Unless otherwise stated, *p* < 0.05 was considered significant.

## 5. Conclusions

High salinity induces the expression of a basic helix–loop–helix transcription factor, *PeGLABRA3*, in the salt-resistant *Populus euphratica*. PeGLABRA3 binds to the promoter of *PePLDδ* to enhance its expression under salt stress. The overexpression of *PeGLABRA3* increases *AtPLDδ* transcription in Arabidopsis, resulting in an increased concentration of phosphatidic acid species under no-salt and saline conditions. We conclude that PeGLABRA3 activated *AtPLDδ* transcription by binding to the *AtPLDδ* promoter region, conferring Na^+^ and ROS homeostasis control via PLDδ- and PA-mediated signaling pathways under salt stress.

## Data Availability

The data presented in this study are available in the article and Supplementary Materials.

## Supplementary Materials

The following are available online at https://www.mdpi.com/article/10.3390/ijms24098208/s1.
