# Supplementary material for: Populus euphratica GLABRA3 Binds PLDδ Promoters to Enhance Salt Tolerance
Source: Int J Mol Sci. 2023 May 3;24(9):8208. doi: 10.3390/ijms24098208 (PMC10179125; doi:10.3390/ijms24098208)
Supplement: Supplementary file 1 [file ijms-24-08208-s001.zip › Supplementary data_corrected.pdf]

Supplemental Figure

```
+ AGAGAGTGAG AATTGGCAAT CCATGTTTTA GGTAACGTCT AAATCTCTTG GAACCATTGG GGTTTACAAG
- TCTCTCACTC TTAACCGTTA GGTACAAAAT CCATTGCAGA TTTAGAGAAC CTTGGTAACC CCAAATGTTC

+ TCTTACTAGC TCACGAACTT AGCCACTTGG GATATCGTGT AACCCGCAGG TCATGATTTA AGAGACAGTG
- AGAATGATCG AGTGCTTGAA TCGGTGAACC CTATAGCACA TTGGGCGTCC AGTACTAAAT TCTCTGTCAC
                                                                 Myc
+ AAAGCTGAGA AAAGGAAGGG ACCGTTTCAA AGTGATCTTC TGTGGATAAC TTTGTGAACA AGGTTTTCTT
- TTTCGACTCT TTTCCTTCCC TGGCAAAGTT TCACTAGAAG ACACCTATTG AAACACTTGT TCCAAAAGAA

+ AATAGATCCT TTTTTCGTGA GTTGTATATT ATTCCAAACT GATCCATAAA TCCATCCAAT CCTCATCCCC
- TTATCTAGGA AAAAAGCACT CAACATATAA TAAGGTTTGA CTAGGTATTT AGGTAGGTTA GGAGTAGGGG

+ CAAAAAGAAA ATAGTATAAA CATAGAAAAA CGAAAACATA TCAGACTTTG GGCTTTTCGT ATGGTTTAGT
- GTTTTTCTTT TATCATATTT GTATCTTTTT GCTTTTGTAT AGTCTGAAAC CCGAAAAGCA TACCAAATCA
                                            MYC            MYC      ARE
+ TTTTGGCTTT TTGCTGTGTT GTTCTATTTC AGAAAGTAAA CATGTGAAAC GGTTCATTTG TAATCCATGA
- AAAACCGAAA AACGACACAA CAAGATAAAG TCTTTCATTT GTACACTTTG CCAAGTAAAC ATTAGGTACT

+ AAGGATTCTT TATGTTACTG CTGTTGCTTC ATTGAGTAGA TACGAATCGA GAATGCCTTT TTTCCTTGTT
- TTCCTAAGAA ATACAATGAC GACAACGAAG TAACTCATCT ATGCTTAGCT CTTACGGAAA AAAGGAACAA
                  ABRE  MYB
+ TCCGACAATT ATCGATTGAC GTGTGACCAC TTTAAAAGTT TAAACAGCTC GACTTTCCAA TATGGGTTTA
- AGGCTGTTAA TAGCTAACTG CACACTGGTG AAATTTTCAA ATTTGTCGAG CTGAAAGGTT ATACCCAAAT
                    G-box
+ TTTCTTGTTT TATCCACACC ATTAAAGAAT GGTTTTTGGG ATTTTTATTT ATGTGATAAT TAATCATTTT
- AAAGAACAAA ATAGGTGTGG TAATTTCTTA CCAAAAACCC TAAAAATAAA TACACTATTA ATTAGTAAAA
                                    ARE
+ TCCAAATTTT ATTTTGTATA TAATAAATGA AGCAAATGTT GGAAAACTAT CCAATGGATG TGGTGGGTTA
- AGGTTTAAAA TAAAACATAT ATTATTTACT TCGTTTACAA CCTTTTGATA GGTTACCTAC ACCACCCAAT
                                    MYC
+ ATATCACCAG ATTCGCATAG CTGGTTTTTG ACTTGTCTTC TTAATTATTT GTCCAGAAAA AGAGAAGAAC
- TATAGTGGTC TAAGCGTATC GACCAAAAAC TGAACAGAAG AATTAATAAA CAGGTCTTTT TCTCTTCTTG
                         ARE
+ TCTTCACATC ATCATTGTCA ACTTTAGCAT TATTGTATTA GCTTTTTATT TCTTTACGTC TACAAAGCTA
- AGAAGTGTAG TAGTAACAGT TGAAATCGTA ATAACATAAT CGAAAAATAA AGAAATGCAG ATGTTTCGAT

+ TTGGTACAAC GTTCTAAAAT CAAATTCGTC ATCAGTAGAT TTTGTAAACT AATTAAGTAA AGTTCAGTGA
- AACCATGTTG CAAGATTTTA GTTTAAGCAG TAGTCATCTA AAACATTTGA TTAATTCATT TCAAGTCACT
                        ABRE
+ TTAAAGAAGC TAGATGAAGA ACGTGTGCAA CGACTCCTCT GAGATCTACA CGGAATAATG TCGTCAGTGA
- AATTTCTTCG ATCTACTTCT TGCACACGTT GCTGAGGAGA CTCTAGATGT GCCTTATTAC AGCAGTCACT
                 G-box    G-Box     G-Box
+ GCAAACAACT CCCATCACGT CGTCCCTCCA CCTGTCCTCT CTTCTCCTTC CTTGCTTGTC TTTCTCTCTC
- CGTTTGTTGA GGGTAGTGCA GCAGGGAGGT GGACAGGAGA GAAGAGGAAG GAACGAACAG AAAGAGAGAG
                 ABRE
+ AAATCATTTC ACCTAAAAAT AATAAATATC TTTCGTTTTC TAAAGAAAAA AAAAAAAAAA ACTTTTCAAA
- TTTAGTAAAG TGGATTTTTA TTATTTATAG AAAGCAAAAG ATTTCTTTTT TTTTTTTTTT TGAAAAGTTT

+ TTCATCTTTG GTTTCTGCAG CAGCAACAAC AACCGAGCCC TGTTCGTTAG GGTTTTCGGT TTTTGTTAGC
- AAGTAGAAAC CAAAGACGTC GTCGTTGTTG TTGGCTCGGG ACAAGCAATC CCAAAAGCCA AAAACAATCG
         ARE                        G-Box
+ TTTTCTTCTT CTTCTTCTTC GTTTCCTTCC ACCTGAATTG TTGTAAC ATG (The start of the coding region)
- AAAAGAAGAA GAAGAAGAAG CAAAGGAAGG TGGACTTAAC AACATTG        AtPLDδ
```

Figure S1. Promoter sequence analysis of *Arabidopsis thaliana AtPLDδ*. The *Arabidopsis thaliana AtPLDδ* promoter was amplified by PCR, and the primer sequences used.for *AtPLDδ* promoter isolation are shown in Supplemental Table S1. The 1,237-bp DNA sequence was analyzed using the PLACE and Plant-CARE databases. The predicted cis-acting elements are as follows: ARE (cis-acting regulatory element essential for the anaerobic induction), ABRE motif (cis-acting element involved in the abscisic acid responsiveness), MYC(cis-acting element in response to drought and ABA), G-box (cis-regulatory element involved in light response). ATG: start codon of the *AtPLDδ* gene. Different colors represent the predicted cis-acting elements in the promoter region.

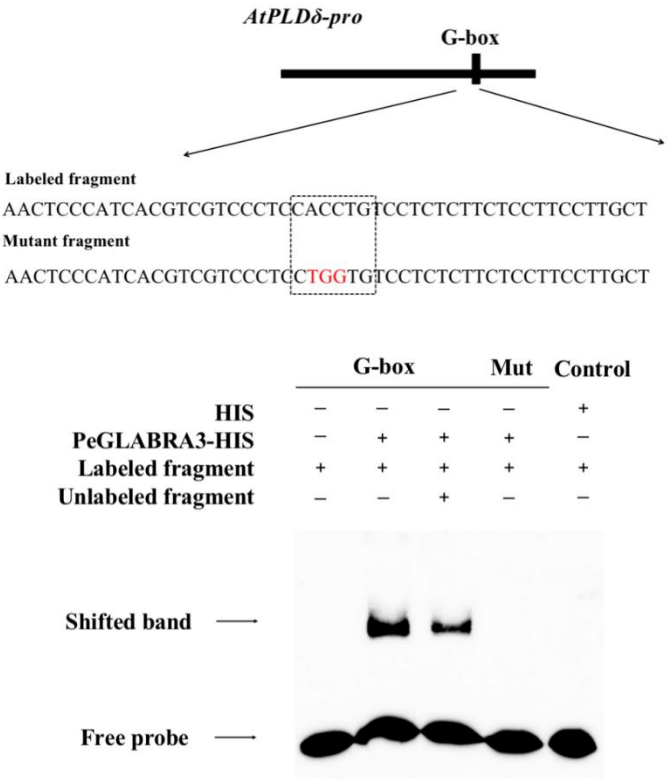

Figure S2. Electrophoretic mobility shift assay (EMSA) verified the interaction of PeGLABRA3 with the *AtPLDδ* promoter region. PeGLABRA3-HIS protein purified from prokaryotic expression was used for in vitro EMSA, while HIS protein was used as a negative control. The mutant probes (Mut, CACCTG to CTGGTG) were used to confirm the binding specificity of G-box to PeGLABRA3. The bases marked in red indicate the mutated bases in the mutant probe. In each panel, "+" and "-" indicate the presence or absence of protein and probe in the loading mixture, respectively. The cold probe concentration was 10× and the concentration of the polyacrylamide gel was 6%. The EMSA experiment was repeated three times and representative images are shown.

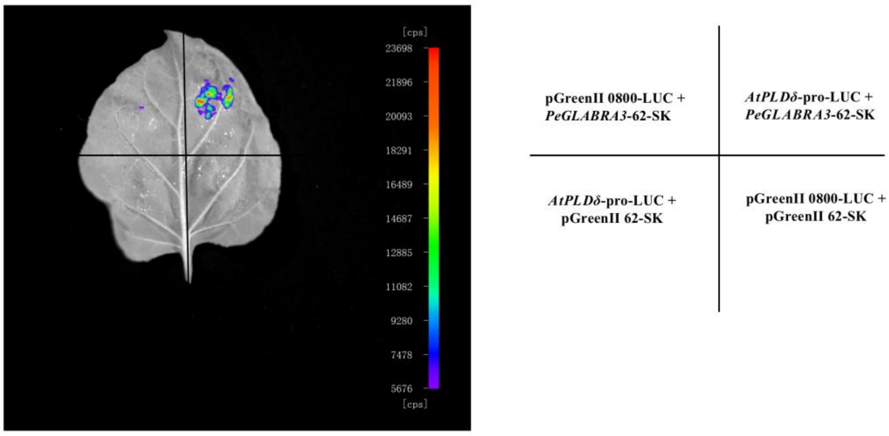

Figure S3. The luciferase reporter assay (LRA) validated the PeGLABRA3 interaction with *AtPLDδ* promoter. *Nicotiana tabacum* leaves were co-transformed with *Agrobacterium* strains containing (1) *PeGLABRA3*-62-SK + pGreenII 0800-LUC, (2) *PeGLABRA3*-62-SK + *AtPLDδ*-pro-LUC, (3) *AtPLDδ*-pro-LUC + pGreenII 62-SK, and (4) pGreenII 0800-LUC + pGreenII 62-SK. The LRA was repeated three times and representative luciferin luminescence images are shown.

Table S1. Primers used in this study.

Gene	Primers
AtSOD	5′-AGGAAACATCACTGTTGGAGAT-3′
	5′-GAGTTTGGTCCAGTAGAGGGAA-3′
AtPOD	5′-CGTGCCCTTCATATTGTTGG-3′
	5′-GACGCCATCAACAACGAGTC-3′
AtCAT	5′-AATATGCTGACGATGAGGATGC-3′
	5′-CAAGAATCAAGGAGGTAGGAGATG-3′
AtSOS1	5′-GTGAAGCAATCAAGCGGAAA-3′
	5′-TGCGAAGAAGGCGTAGAACA-3′
AtSOS2	5′-GGGCAGTTATGTAGCGGAGA-3′
	5′-TTTCACCAGCAGCCTTTCTT-3′
AtACTIN2	5′-GGTAACATTGTGCTCAGTGGTGG-3′
	5′-AACGACCTTAATCTTCATGCTGC-3′
AtPLDδ	5′-GTAGCTGCTAAAGCTTACGA-3′
	5′-AAAGAACAAACAACAAATGG-3′
AtPLDδ promoter	5′-AAGAGAGTGAGAATTGGCAATCCATG-3′
	5′-GGTTACAACAATTCAGGTGGAAGGAA-3′
PePLDδ	5′-TGATGGGCCAGCTGCATATGAT-3′
	5′-AGGTGGAACTATTGTGGTTCCATCC-3′
PePLDδ promoter	5′-TAAAAATAATATTTTTTATTTTTT-3′
	5′-TTTATTGGGCGGGATTTTTCAGAG-3′
PeGLABRA3	5′-GACAAAGAGCTTGCATGTGCCAC-3′
	5′-CAAACTTTGCATTTGGGAAGCTCC-3′
PeACT7	5′-ATTGGCCTTGGGGTTAAGAG-3′
	5′-CACACTGGAGTGATGGTTGG-3′

Table S2. Accession numbers of GLABRA3 orthologs used in multiple sequence alignment and phylogenetic analysis.

Sequence name	Accession number
AtGLABRA3	NP_680372.1
NtGLABRA3	NP_001313104.1
PeGLABRA3	XP_011029287.1
PtGLABRA3	XP_024445100.1
GmGLABRA3	XP_003532789.1
OsGLABRA3	XP_015616671.1
